# Analysis of gene expression in filamentous cells of *Candida albicans* grown on agar plates

**DOI:** 10.14440/jbm.2018.211

**Published:** 2018-01-23

**Authors:** Paul E. Creger, Jill R. Blankenship

**Affiliations:** Biology Department, University of Nebraska Omaha, Omaha, NE 68182, USA

**Keywords:** *Candida albicans*, filamentation, RNA extraction, qRT-PCR, RNAseq

## Abstract

*Candida albicans* (*C. albicans*) is a commensal organism of the human gastrointestinal and genitourinary tracts. *C. albicans* is also a major human pathogen, causing disease ranging from cutaneous infections to lethal systemic disease. The ability of this fungus to switch between yeast and filamentous forms of growth has long been linked to its pathogenesis. Filamentation can be induced by a variety of distinct environmental cues and can occur in either liquid or solid media. While some evidence suggests that there are differences between filamentation in solid and liquid media, gene expression analysis of filamentation in *C. albicans* has focused strictly on cells grown in liquid media. We have developed a method for analyzing gene expression of filamentous cells grown on solid induction media at early stages of filamentation, establishing cell plating densities, ideal collection times, and collection techniques. We have also demonstrated the utility of the approach not only in qRT-PCR assays, but high-throughput RNAseq assays as well. These assays will allow for comparison studies of *C. albicans* filamentation initiation in solid and liquid media.

## INTRODUCTION

The pathogenic fungus *Candida albicans* (*C. albicans*) is a common commensal organism in the gastrointestinal and genitourinary tracts of humans. It can also be pathogenic, with infections ranging from cutaneous infections of the oral and genital mucosa to lethal systemic disease. *C. albicans* and other *Candida spp*. infections are a leading cause of systemic infections in hospital settings and mortality rates are high (~40%) [1].

*C. albicans* is a dimorphic fungus that can grow in a variety of forms from yeast-like cells to true filaments. The ability to transition between the yeast and filamentous forms is vital to pathogenesis [2-4] and is the most heavily studied aspect of *C. albicans* biology. Several factors are known to induce filamentation, including high temperature (37°C), neutral or slightly basic pH, nutrient deprivation, and exposure to serum. Filamentation can occur in liquid media, solid media, and within embedded conditions. Phenotypic assays are generally performed in a variety of media conditions, but very little effort has been focused on comparisons of gene expression between the conditions.

While phenotypic assays can span a variety of conditions, gene expression studies of *C. albicans* comparing filamentous and yeast cells have focused on gene expression in filamentation of cells in liquid media. There are a number of reasons filamentation studies have focused on expression in liquid conditions. There is a general perception that recovery of cells would not be sufficient for high-throughput studies from standard agar plates. This perception is exacerbated by the fact that filamentous cells are adherent to surfaces and will invade agar surfaces, making recovery of cells difficult. In addition, filamentation in *C. albicans* is inhibited by quorum sensing factors, restricting the number of cells that can be spread on solid surfaces. We have developed a method for harvesting *C. albicans* filamentous and yeast cells from solid media for RNA extraction that can be used in qRT-PCR and RNAseq studies. We identified cell density conditions appropriate for filamentation and time points at which cells could be recovered. As a proof of principle, we extracted RNA from wild type cells grown in 5 solid conditions and demonstrated the utility of the RNA in qRT-PCR and RNAseq analysis.

## MATERIALS AND METHODS

### Strains and media

The *C. albicans* strains SC5314, P87, and P76067 [5] were grown on yeast-extract-peptone-dextrose (YPD) medium (10 g yeast extract, 10 g peptone, 2% glucose, and 16 g agar in 1 L of water), spider medium (10 g D-mannitol, 10 g nutrient broth, 2 g K_2_HPO_4_, and 16 g agar in 1 L of water), 10% FBS media (100 ml fetal bovine serum, 2% dextrose, and 16 g agar in 1 L of water), Lee’s media [6], and RPMI-MOPS media (1× RPMI solution, 2.1 mM L-glutamine, 0.165 M MOPS buffer pH 7.2, 1.8% glucose, and 16 g agar in 1 L of water). Agar plates were poured into 100 mm (20–25 ml media) or 150 mm petri dishes (45–50 ml media) (Fisher). Cells were grown at 30°C for yeast-like growth, and at 37°C for filamentous growth. Plates were placed in the incubator in a single layer.

### Filamentation assay

A single colony of SC5314 was picked from a YPD agar plate struck from a glycerol frozen stock and incubated overnight in liquid YPD media at 30°C with shaking. One ml aliquots of this overnight culture were spun down at high speed in a microcentrifuge, and washed twice with 1 ml of phosphate buffered saline (PBS) pH 7.4. The washed cell pellets were resuspended in 1 ml PBS. 140 μl of washed cells (approximately 39 million cells) and 200 μl of sterile water were spread with 3 mm glass beads onto YPD, spider, 10% FBS, Lee’s, and RPMI-MOPS agar plates in the larger plates. Smaller plates were spread with varying cell volumes indicated in the text. Cells were incubated at 37°C (all media) with an additional yeast control on YPD at 30°C. For the dilution assay, cells were recovered from the plate surface in a similar manner to the cell harvesting below.

### Cell harvesting

Following incubation and imaging, 2 ml of sterile, autoclaved nanopure water was added to the top of the agar plate. A sterile, disposable cell scraper was scraped across the surface of the plates to liberate cells from the agar surface and to collect the water/cell slurry at a collection point on the plate. The water/cell slurry was pipetted into microcentrifuge tubes and quickly spun down at full speed in a microcentrifuge. The supernatant was poured off and the cell pellets were frozen at –80°C.

### RNA purification and DNase treatment

RNA was collected from frozen cell pellets using the RNeasy kit (Qiagen) with minor modifications. Cell pellets were resuspended in the supplied resuspension buffer with 10 μl/ml beta-mecaptoethanol and moved to a 2 ml screw-cap tube. Cells were mechanically disrupted using 450 nm glass beads in a mini-bead beater (RPI), with three 1 min bead beating pulses and 2 min rests. The resulting supernatant was cleared of cell debris and RNA was precipitated with 70% EtOH. The cell supernatant/EtOH mix was placed into the purification column, washed, and eluted with 100 μl nuclease-free water. Residual genomic DNA was removed from the RNA samples during washing using a Qiagen RNase-free DNase on-column removal kit.

### qRT-PCR gene expression analysis

cDNA was generated from approximately 3 μg of DNA-free RNA per sample using the Maxima First Strand kit (Thermo Fisher) following the manufacturer’s instructions. This kit uses a mixture of oligo dt and random primers to prime DNA synthesis. An additional 3 μg sample of RNA was incubated without the kit’s enzyme mix to serve as a no-rt control. Following cDNA generation, 30 μl of nuclease-free water was added to each cDNA and no-rt control reaction for a final volume of 50 μl. One μl of cDNA, 10 μl of 2× Dynamo Flash SYBR green master mix (Fisher), 2 μl of each primer (**Table 1**), and 5 μl of nuclease-free water was added to each qRT-PCR tube. The reactions were run on a Qiagen Rotogene Q using cycles with an initial denaturation at 95°C, followed by 40 cycles of 15 s denaturing at 95°C and 30 s annealing/extension at 60°C. SYBR imaging was taken during the annealing/extension step. TDH3 expression was used as a control to normalize expression between conditions.

### RNAseq gene expression analysis

RNASeq libraries were generated beginning with 1.8 ng of total RNA following standardized protocols with the TruSeq RNA v2 kit (Illumina, San Diego). Libraries were diluted to a concentration of 6.0 picomoles and sequenced on a HiSeq2500 and 100 bp single reads were generated.

All 8 Candida chromosomes were downloaded from NCBI, and annotation was downloaded into a gff3 file, and transformed into a gtf file using gffread. Fastq files were generated using the bcl2fastq software, version 1.8.4. The fastq files for each sample were analyzed using the Tuxedo pipeline in order to find differentially expressed genes. Read alignment was performed using tophat version 2.0. FPKM values were calculated with cufflinks 2.2. The cuffmerge and cuffdiff software were used to calculate fold change values between sets of samples. A *P* value of 0.05 was used to differentiate between statistically significant and insignificant gene expression changes.

### Cell imaging and statistics

Images of cells grown on solid media were taken using an EVOS FL inverted microscope with a 10× objective using the 10× phase filter. Images were taken through the bottom of the agar plate at the indicated times. For the dilution assay, harvested cells were aliquoted onto glass slides and imaged on a Zeiss Axiovision microscope with a 20× objective. Assays were run in triplicate and cells were manually counted using the cell counter plug-in on ImageJ. Student’s *t* tests were performed to analyze statistical significance of values.

## RESULTS

### Plating density impacts filamentation

**Figure 1.**
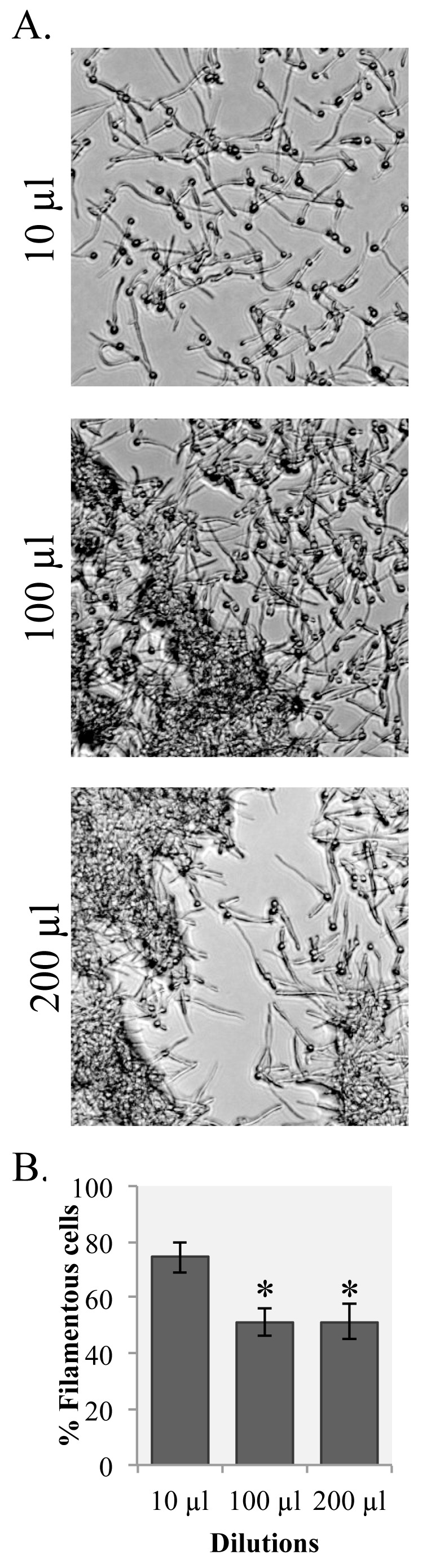
Plating density impacts filamentation. Washed overnight cultures of wild type *C. albicans* strain SC5314 were spread onto RPMI-MOPS plates at the indicated volumes. For volumes less than 200 μl, sterile water was added to the surface of the plate to bring the plating volume to 200 μl. Images are representative pictures from three independently-tested plates. **A.** The plates were allowed to incubate at 37°C for 3 h and cells were then imaged on the plate surface. **B.** The percentage of filamentous cells was calculated for each dilution by averaging filamentous cells recovered from three agar plates. Error bars represent standard error from the mean. Starred values are significantly different from the 10 μl dilution (*P* < 0.01) as measured by a Student’s *t*-test.

**Figure 2.**
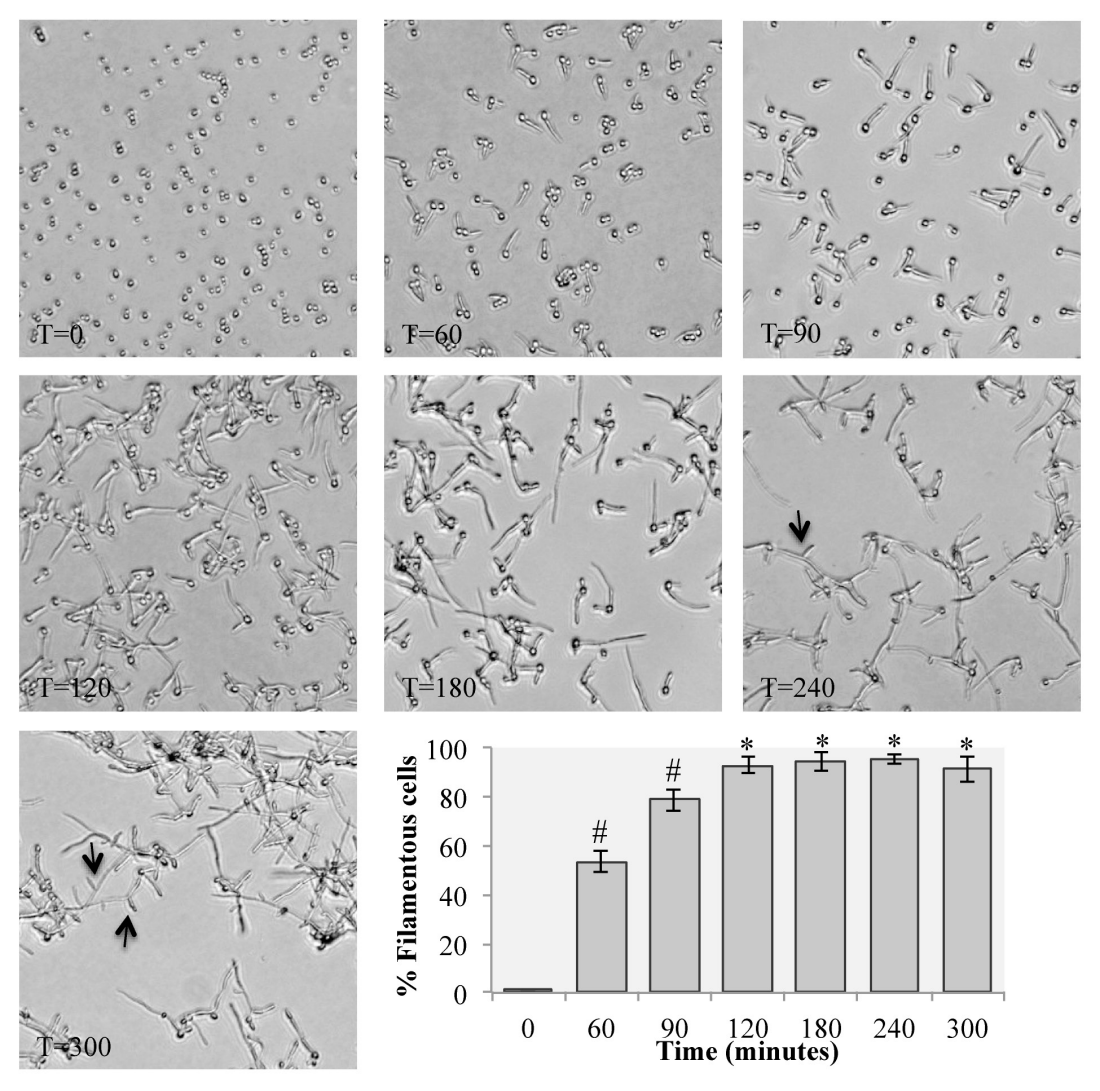
Filament development over time on solid agar plates. Ten μl of washed overnight cultures of wild type *C. albicans* strain SC5314 were spread onto RPMI-MOPS plates and incubated at 37°C for the indicated times (in minutes). Arrows at 240 and 300 min indicate branching of the filamentous cells. The inset graph represents the percentage of filamentous cells at each time point that were counted on from images taken from 3 plates. Starred values are significantly different from the 0 time point (*P* < 0.001) as measured by a Student’s *t*-test. Hash-marked values are significantly different from the 0 time point and from the previous time point (*P* < 0.005).

Overnight cultures of *C. albicans* wild type SC5314 cells grown at 30°C in YPD liquid media were washed twice with PBS to remove YPD and quorum sensing molecules, such as farnesol and tyrosol, that might impact filament development [7,8]. Our first assay was to determine an appropriate concentration of cells for maximal filamentation. Ten μl, 100 μl, or 200 μl of washed cells were plated onto 100 mm RPMI-MOPS plates and monitored for 3 h (**Fig. 1A**). Plating density had a significant effect on filamentation, and the higher densities had a negative impact on the percentage of plated cells filamenting (**Fig. 1B**). The percentage of filamentous cells recovered from the 10 μl plates (**Fig. 1B**) is lower than the percentage of filamentous cells counted directly from plates in **Figure 2** (comparison with 180 min), suggesting that scraping and counting underestimates the percentage of filamentous cells at all densities. This is likely because the filamentous cells, but not yeast cells, adhere to one another in solution and are difficult to count on slides when clumped together.

### Timing of filamentation on agar plates

For our second set of experiments, we assayed filamentation over time to identify a time-point at which most, if not all, cells were filamentous and cells could be recovered from the agar surface. In this assay, cells were monitored over 300 min, and sample scrapings were taken on plates after filamentation was observed on plates. The washed cells, at plating (T = 0), were clearly yeast cells and largely remained so at 60 min following incubation on the RPMI-MOPS plates at 37°C (**Fig. 2**). A few instances of hyphal or pseudohyphal development were observed at this time. The majority of cells were filamentous from 120 min post-incubation to the end of the experiment at 300 min (**Fig. 2**). At the final time-point of 300 min, we observed significant branching of the filamentous cells (**Fig. 2A**, arrows). In the cell scraping assays, cells were readily scraped at 90, 120, and 180 min post-incubation, but cells appeared to be fairly imbedded at later time-points and few cells could be scraped from the surface of the agar plates (data not shown).

### Divergent *C*. *albicans* strains have distinct filamentation phenotypes

**Figure 3.**
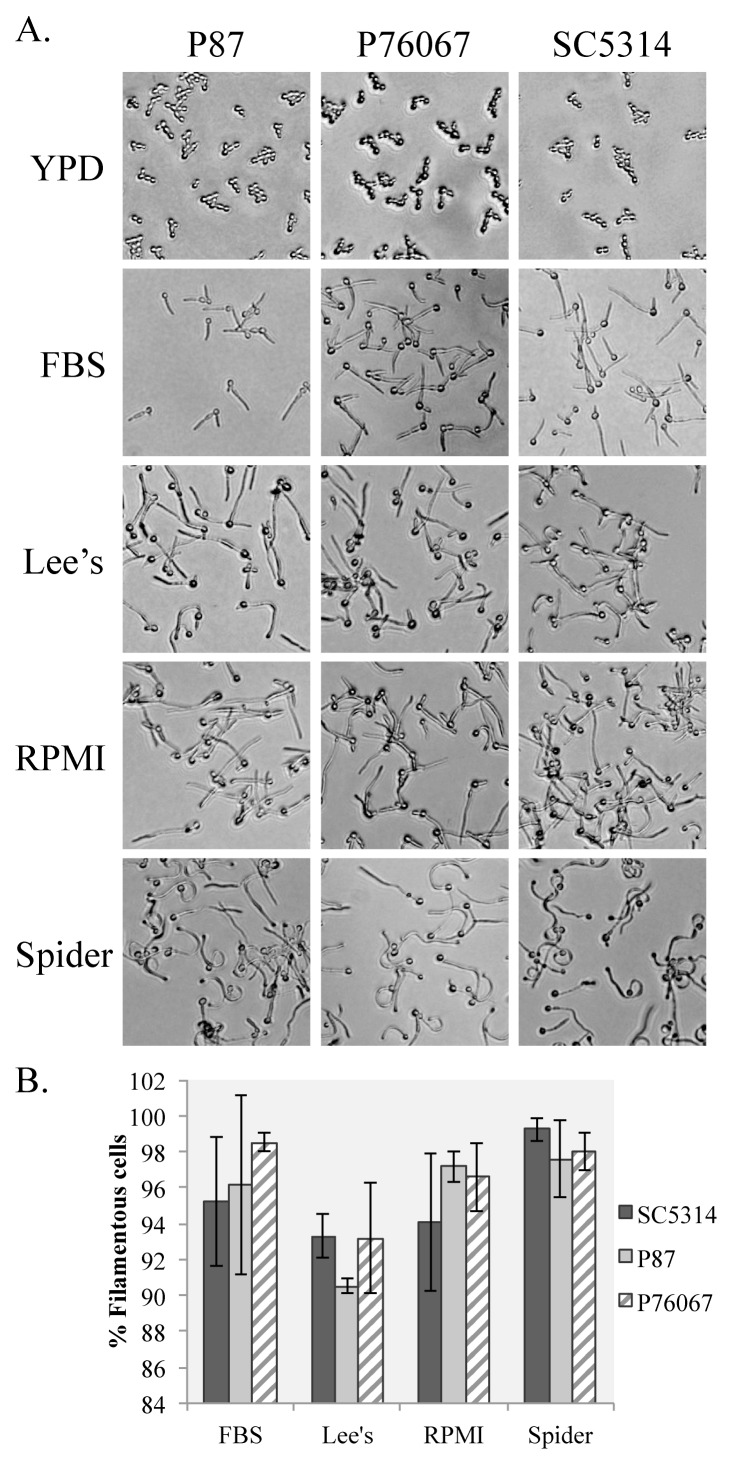
Divergent *C. albicans* wild type strains exhibit distinct filamentation phenotypes on inducing media. **A.** Washed overnight cultures of wild type *C. albicans* strains P87, P76067, and SC5314 were spread onto inducing media (FBS, Lee’s, RPMI, and Spider) and the non-inducing media (YPD). Plates were incubated for 180 min at 37°C for the inducing media and 30°C for the non-inducing media and then imaged. **B.** The percentage of filamentous cells was calculated for each strain by averaging filamentous cells recovered from three agar plates. Error bars represent standard error from the mean. No values had a statistically significant difference from the SC5314 value measured within the same condition.

The strain chosen for the majority of this work, SC5314, is one of the most commonly used *C. albicans* strain backgrounds. We wanted to investigate how transferable our assay is to wild type *C. albicans* backgrounds divergent from SC5314. We therefore looked at the filamentation of strains P87 and P76067 in each solid condition at 180 min. These strains belong to different clades of *C. albicans*, are genetically distinct from SC5314, and should give a broad indication of how divergent strains may behave in solid filamentation conditions [5]. As expected, these strains did not filament in solid YPD media at 30°C and were fully filamentous in 10% FBS (**Fig. 3**). There was some slight variability in filamentation on the varied media between the strain types, but none of the variation reached statistical significance (**Fig. 3**). However, variations will likely exist in some non-SC5314 strains and will need to be examined prior to any gene expression analysis.

### Cell harvesting for RNA extraction

The goal of investigating filamentation on agar plates was to identify a strategy that could be utilized to harvest cells for gene expression assays. As a proof of principal, wild type SC5314 *C. albicans* cells were incubated on YPD, spider, 10% FBS, Lee’s, and RPMI-MOPS agar plates for 180 min at 37°C (all plates) or 30°C (YPD-only). For this assay, plate size was scaled up from 100 mm petri dishes to 150 mm petri dishes and the volume of washed overnight cell culture added to the plate was increased accordingly. Cells were imaged following incubation (**Fig. 3**, SC5314), harvested by adding 2 ml of room temperature, sterile water to the plates, and gently scraping with a sterile cell scraper. Water was added to the surface of the plate prior to scraping because this increased the number of cells collected from the plate surface (data not shown). Cells were collected in microcentrifuge tubes, spun down, and the pellets were frozen at –80°C. RNA was isolated from the cell pellets and assessed by spectroscopy. RNA quality, as measured by 260/280 ratio, were all above 2.0 with an average of 2.17, and RNA concentrations for all samples were relatively abundant except for RNA collected from cells grown in 10% FBS (**Table 2**).

### Gene expression determination

**Figure 4.**
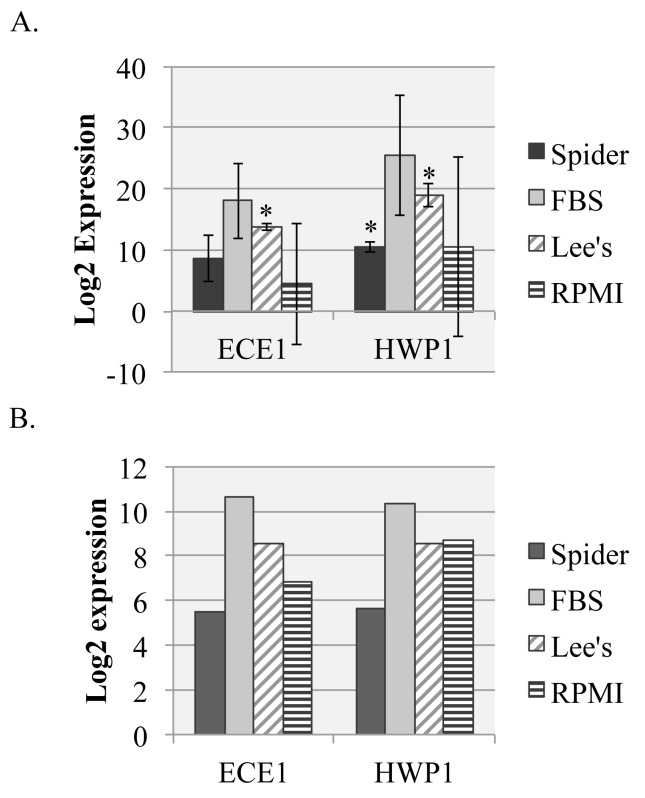
qRT-PCR and RNAseq expression analysis of hyphal-specific genes. **A.** The expression of hyphal-specific genes *ECE1* and *HWP1* in SC5314 cells grown on spider (dark bars), 10% FBS (light bars), Lee’s (diagonal striped bars), or RPMI (horizontal striped bars) solid media were examined by qRT-PCR. *TDH3* (not shown) was used as to normalize gene expression between samples and expression of cells grown in YPD at 30°C was used as a normalizing condition. Error bars represent standard deviation from the mean of three biological replicates. Starred values are significantly different from the control strain (*P* < 0.05) as measured by a Student’s *t*-test. **B.** The expression of hyphal-specific genes *ECE1* and *HWP1* in SC5314 cells grown on the indicated solid media were examined by RNAseq analysis. Expression levels for each sample were normalized to expression levels in cells grown in YPD at 30°C. Samples were tested in biological triplicates. All samples were significantly different from control values (*P* < 0.001).

Two assays were initiated with RNA collected from the cells grown on plates, qRT-PCR analysis and RNAseq analysis. The goal of the qRT-PCR analysis was to determine whether gene expression could be reliably detected from cells grown on solid media and to examine the expression of genes known to be upregulated in filament-inducing liquid conditions. The expression of two genes, *HWP1* and *ECE1*, and the control gene *TDH3* were measured by qRT-PCR from these samples. *HWP1* and *ECE1* are highly upregulated in liquid filamentation conditions and specific to *C. albicans* hyphal cells [9-11]. We anticipated observing upregulation in solid conditions as well. With the exception of *HWP1* expression in solid spider media, we observed the expected upregulation of *HWP1* and *ECE1* in solid filamentation conditions (**Fig. 4A**). Thus, gene expression was reliably detected and appeared to show a filamentous hallmark.

The goal of the RNAseq analysis was to determine whether RNA extracted from cells grown on solid plates was sufficient for analysis using this method. RNA quantity from single large-plate assays with fairly dilute cell plating was more than sufficient for RNAseq library preparation (**Table 2** and **Table S1**). The quality reading from our RNA samples varied between runs from 6.5–9.7, with average quality scores above 8 in our triplicate assays (measured by RQN in **Table 3**). RNA quality number (RQN) scores are equivalent to more widely used RIN (RNA Integrity Number) scores. While values of 6.5 are lower than ideal, qRT-PCR analysis of housekeeping gene expression in samples with quality scores above 6 vary little from samples with higher quality scores [12]. The lower reads in samples below 7.0 were likely due to issues in sample prep rather than recovery from solid plates. Reads ranged from ~16–41 million reads per sample and following mapping, less than 8% of reads were unmapped. We looked at the expression of *ECE1* and *HWP1* to determine whether there was correlation between the qRT-PCR data and the RNAseq data. The expression data, measured in triplicate biological replicates, mirrors the data from the qRT-PCR experiment, with similar values and expression trends in each condition (**Fig. 4B**).

## DISCUSSION

*In vitro* studies of gene expression in microbes largely focus on cells grown in liquid culture due to the ease of cell collection and the ability to easily increase culture volume, and thus cell numbers. This is particularly true for investigations that could be impacted by cell concentration, like filamentation in *C. albicans*. Our initial assays to determine appropriate concentrations of plated cell volumes suggested that dense plating did indeed negatively impact filamentation. This put a significant limitation on the number of cells that could be collected per plate and how the cells could be recovered, necessitating the addition of water to the plate surface prior to cell collection.

Despite the low plating density required for our assay, we found that we could collect enough cells from a single 150 mm plate to harvest RNA for qRT-PCR and RNAseq assays. Within an acceptable range, we did observe variability in the total RNA that could be collected from cells on each plate, with cells grown on FBS and Lee’s media yielding consistently low amounts, while non-inducing conditions consistently yielded the highest amount of RNA (**Table 2**). We hypothesize that slower growth on FBS and Lee’s media, rather than increased cell adherence to the plate surface, may have contributed to differences in cell, and thus RNA, recovery. In support of this hypothesis, we did not see major differences in adherence gene expression between filamentation-inducing samples in our RNAseq analysis (data not shown), suggesting cells were not adhering more tightly to agar surfaces in FBS and Lee’s media.

Expression analysis demonstrated the efficacy of RNA harvested from cells grown on solid agar plates for both qRT-PCR and RNAseq analysis. Both *ECE1* and *HWP1*, genes known to be highly upregulated in hyphal conditions in liquid media, were upregulated in solid media in both the qRT-PCR and RNAseq analysis, suggesting that this approach is capturing filamentous gene expression from cells grown on the surface of agar plates. This method can be used to probe gene expression differences that may exist between filamentous cells grown in liquid culture and those grown on the surface of agar plates. This approach would also be amenable to recovery at earlier time points to capture a time course of gene expression in early filamentation response. Recovery at later time points, as filaments mature, will require additional work to recover cells due to the difficulty of recovering these highly adherent cells from plate surfaces.

## Supplementary Material

Supplementary information **Table S1**. Extended RNAseq quality data.
Supplementary information of this article can be found online at
http://www.jbmethods.org/jbm/rt/suppFiles/211.

## Figures and Tables

**Table 1.** Primers used in this study.

Gene	Forward primer	Reverse primer
*TDH3*	AAATCGGTGGAGACAACAGC	TGCTAAAGCCGTTGGTAAGG
*ECE1*	GAGATGGCGTTCCAGATGTT	TACTGAGCCGGCATCTCTTT
*HWP1*	TCTACTGCTCCAGCCACTGA	CCAGCAGGAATTGTTTCCAT

**Table 2.** RNA quantity and quality.

	RNA ng/μl (260/280)		
Treatment	Set 1	Set 2	Set 3
YPD-30°C	678.0 (2.19)	350.6 (2.21)	983.0 (2.19)
YPD-37°C	534.2 (2.16)	986.6 (2.20)	1092.6 (2.20)
FBS	45.8 (2.04)	51.8 (2.11)	97.9 (2.13)
Lee’s	183.8 (2.14)	143.6 (2.15)	315.2 (2.18)
RPMI	444.5 (2.16)	743.3 (2.19)	564.5 (2.20)
Spider	489.1 (2.15)	1098.5 (2.19)	963.4 (2.19)

**Table 3.** RNAseq data quality.

Condition	Reads^1^	Unmapped reads^1 (%)^	RQN score^2^
YPD (30°C)	28.35	1.50 (5.30)	8.20
FBS	32.72	1.79 (5.47)	9.00
Lee’s	29.63	1.47 (4.96)	8.43
RPMI	30.45	2.35 (7.71)	9.03
Spider	29.28	1.56 (5.34)	8.67

^1^Data shown is an average of three independent experiments in millions of reads.

^2^The scale is from 0–10, with higher numbers indicating higher RNA quality.
